# Supplementary material for: Sodium, Potassium and Iodine Intake, in a National Adult Population Sample of the Republic of Moldova
Source: Nutrients. 2019 Nov 28;11(12):2896. doi: 10.3390/nu11122896 (PMC6950169; doi:10.3390/nu11122896)
Supplement: Supplementary file 1 [file nutrients-11-02896-s001.pdf]

Table S1. Characteristics of the excluded participants and comparison with those included in the study.

Variable	Included Participants (n = 858)	Excluded Participants* (n = 436)	*p*-value
Age (years)	48.5 (13.8)	48.6 (14.2)	0.88
Height (cm)	166.7 (8.8)	166.6 (8.7)	0.83
Weight (kg)	78.2 (15.8)	75.7 (15.4)	0.009
B.M.I. (kg/m^2)	28.1 (5.4)	27.3 (5.5)	0.011
Waist circumference (cm)	92.6 (15.1)	91.0 (15.5)	0.09
Hip (cm)	104.2 (13.4)	101.2 (12.7)	0.001
Systolic BP (mmHg)	134.3 (21.2)	133.2 (19.1)	0.38
Diastolic BP (mmHg)	86.8 (11.9)	85.1 (10.9)	0.013
Pulse rate (b/min)	76.2 (9.5)	76.5 (9.5)	0.61
Hypertension (%)	45.5	40.2	0.08

* Participants without complete urines (see Figure 2)

Text S1. Data Collection

The examination was performed in a quiet and comfortable room, with the participants who were not allowed to smoke, exercise, eat, and consume caffeine and to have a full bladder for 30 minutes before measurements. The survey was carried out in three steps: a) questionnaire survey, b) physical measurements, and c) 24 h urine collections.

The questionnaire (face-to-face interview, adapted version of the WHO STEPS Instrument for NCD Risk Factor Surveillance) [35] was used to collect data on respondent's demographic and socio-economic status (by occupation and educational attainment); diet, frequency of high salt food consumption, fruit and vegetable consumption, knowledge attitudes and behaviour on dietary salt, history of high BP, diabetes and CVDs, and lifestyle advice.

Anthropometric indices, BP, and heart rate were measured in all participants. Height was measured in cm and body weight was measured in kg using a digital electronic device (body scale with a height laser gauge) (Growth Management Scale). Body mass index (BMI) was calculated as weight (kg) divided by height squared (m^2). Waist and hip circumferences were measured by myotape, a non-stretch tape with mm precision as described elsewhere [32,33]. Systolic and diastolic BP and heart rate measurements were taken three times in the right arm on a sitting position, using a universal cuff and automatic BP and heart rate monitors (Boso Medicus Uno, Bosch+Sohn GmbH, Jungingen, Germany). The first measurement was ignored, the mean of the second and third measurements being taken for analysis. The measurements were taken after the participant had rested for 15 minutes and each with three minutes of rest between the measurements (maximum deviation of cuff pressure measurement ± 3 mmHg and of pulse rate display ± 5%). Hypertension is defined as systolic and/or diastolic BP $\geq$ 140/90 mmHg or regular antihypertensive treatment [36].

A single 24 h urine collection was obtained from the participants. Each participant was given a leaflet with explanations along with the necessary equipment and a record sheet on which participant noted the start and the finish times of their urine collection, any missed urine aliquots and any medication taken during the collection. The participants were carefully instructed on urine collection methodology [32,33]. In an effort to minimize bias, participants were also requested not to change their diet before or during the day of the urine collection. The first void upon waking on the day of collection was discarded. The urine volume of the 24h collection was measured by field team-members and a urine sample was stored in a cool place for a maximum of 24h until transportation to the laboratory. Sodium, potassium, and creatinine determinations were carried out immediately [37,38]. Sodium and potassium concentration in the urine samples were determined using an Ion selective electrode with a Beckman Coulter Synchron CX5PRO system and

expressed in mmoL/L [37]. Creatinine concentration was determined through the creatinine (urinary) Jaffé kinetic method and expressed in mg/dL [38]. These determinations were carried out at the ICS Medical Laboratory Synevo SRL in Chişinău. Urinary iodine was measured separately at the National Agency for Public Health of the Republic of Moldova using the ammonium persulfate digestion method with spectrophotometric detection by Sandell–Kolthoff reaction, expressed as mcg/L [39]. Iodine determinations in table salt were carried out by the titration method [40].
